# The Mediating Effect of Running Biomechanics, Anthropometrics, Muscle Architecture, and Comfort on Running Economy Across Different Shoes

**DOI:** 10.1111/sms.70087

**Published:** 2025-06-05

**Authors:** Bas Van Hooren, Raf Copier, Sissel Pedersen, Zoi Balamouti, Kenneth Meijer

**Affiliations:** ^1^ Department of Nutrition and Movement Sciences, NUTRIM Institute of Nutrition and Translational Research in Metabolism Maastricht University Medical Centre+ Maastricht the Netherlands

**Keywords:** biomechanics, correlation, energy cost, running economy, shoe

## Abstract

Advancements in shoe technology can improve running economy (RE). However, the effects of advanced footwear technology (AFT) have typically been investigated in one specific shoe brand and at relatively high speeds. Moreover, there is often considerable variability in the response to different running shoes. This study, therefore, investigated the effect of five different shoes (two with AFT, one standard shoe, one traditional racing flat, and participants' own shoes) on RE at a speed representative of recreational runners. Further, it also explored whether spatiotemporal running metrics, anthropometrics, muscle architecture, or comfort mediated the effect of shoes on RE. Forty‐one (31 male) recreational runners ran at 10 km∙h^−1^ in five different shoes while running biomechanics and gas exchange data were collected. Linear mixed models were used to compare RE across different shoes. Correlations were computed between the difference in RE and (difference in) spatiotemporal running metrics, anthropometrics, muscle architecture, or comfort to explore mediating effects. RE was significantly better in the AFT shoe one compared to other shoes by 2%–4%, with the enhancement relative to the other AFT being non‐significant (2%). No spatiotemporal, anthropometric, muscle architectural, or comfort outcome was consistently significantly associated with relative RE. However, a longer contact time, shorter flight time, and higher duty factor showed consistent small‐to‐moderate non‐significant associations with better relative RE. In conclusion, AFT technology can enhance RE at speeds typical for recreational runners, although with variable magnitude across different brands. Further, anthropometrics, spatiotemporal metrics, muscle architecture, nor comfort strongly influenced the effect of shoes on RE.

## 1 Introduction

Running continues to be one of the most popular sports, with, for example, 48 million individuals in the United States running at least once a week in 2023 [1]. This high popularity is also reflected in the popularity of major race events, where individuals often aim to achieve their best performance. Strategies that enhance running performance are therefore of high interest to runners aiming to improve their performance, as well as to coaches, sports scientists, and sports equipment manufacturers.

Advancements in footwear technology (e.g., highly compliant and resilient midsole, embedded longitudinal stiffening elements such as a carbon plate, curved sole geometry) have been shown to improve running performance [2, 3, 4, 5], primarily through improvements in running economy (RE; i.e., by reducing the oxygen or energy required to run at a given steady‐state speed) [4, 6, 7]. Studies comparing advanced footwear technology (AFT) with traditional racing shoes, for example, demonstrated improvements in RE by 2.8%–4.4% relative to traditional racing flats at speeds typical for highly trained and elite runners (14–18 km∙h^−1^) [6, 8, 9]. Recent studies also show improvements in RE at speeds more typical for recreational runners (10–12 km∙h^−1^) [4, 10, 11, 12], albeit often with smaller magnitudes than higher speeds [10, 12, 13].

An important finding of footwear studies is that the response to footwear is highly individual [4, 7, 13, 14, 15, 16, 17], with some individuals showing substantial improvements (e.g., by ~11.4% [13]), whereas others show worse RE (e.g., by ~11.3% [13]) when running in shoes with AFT compared with traditional racing shoes. Although this variability could at least partially reflect measurement error in the gas exchange system [18] and trial‐to‐trial biological variability [19], studies that have averaged multiple counterbalanced measurements of the same individual to reduce measurement error and biological variability still report variable –albeit smaller– effects between different individuals wearing the same shoes, with some individuals showing up to ~7% benefit and others showing no benefit [6, 8, 11, 19]. This variability has led several researchers to recommend an individualized approach to running shoe prescription [4, 13, 16].

The robust effect of shoes with AFT features on RE at the group level, alongside a highly variable response at the individual level, suggests the effect is not solely a direct mechanical effect, resulting from a change in footwear properties, but that it may be mediated by biomechanical and anthropometrical factors [20], that partly explain the variability in responses to the same shoe. Some runners may, for example, increase their leg stiffness more than others in specific shoes, thereby possibly increasing the beneficial effect of the shoes on RE because of the relationship between leg stiffness and RE [21]. Similarly, an anterior shift in the center of pressure induced by shoes with a longitudinal stiffening element [22] may be more beneficial in individuals with certain anthropometric characteristics (e.g., specific Achilles tendon moment arm, foot length, calf muscle architecture) because of the alterations in ankle extensor force production and shortening velocity requirements. These mediating effects may be explored by investigating the relationship between the relative increase or decrease in biomechanical or anthropometric factors and RE across different shoes. Few studies have, however, explicitly investigated the possible mediating effects of biomechanical or anthropometric factors on RE across multiple shoes, with the few studies that have done such analysis often using only two shoes and relatively small sample sizes (*n* < 20) [7, 10, 23].

This study therefore aims to (1) evaluate the impact of two AFT shoes on running economy at speeds typical for recreational runners and (2) investigate whether biomechanical, anthropometric, or muscle architectural factors mediate the effect of shoes on RE across a range of different shoes, including two different shoe brands with AFT features, one traditional racing shoe, one ‘standard’ neutral training shoe, and the participant's own shoes. Moreover, given that previous research has shown higher shoe comfort to be associated with better RE [24], we also investigate whether comfort mediates the effect of running shoes on RE.

## 2 Methods

### 2.1 General Study Design

This study used a randomized crossover design whereby all participants completed one test session where they ran on a treadmill in five different shoes while three‐dimensional ground reaction forces, full‐body kinematics (not reported in this study), and respiratory gases were collected. All participants were instructed to avoid strenuous activity for 36 h, alcohol for 24 h, caffeine for 6 h, and a heavy meal 1 h before the session.

### 2.2 Participants

Forty‐one recreational runners (31 men, 10 women, mean ± SD age 24.4 ± 4.8 years; body height 1.81 ± 0.09 m; body mass 77.3 ± 8.8 kg; weekly running distance 21.8 ± 18.0 km; runs per week 2.88 ± 1.48 times; running experience 5.1 ± 4.5 years), who were free of any minor musculoskeletal injuries for 1 month and of moderate musculoskeletal injuries for 3 months, participated. Moderate injuries were defined as injuries that required at least 1 week of rest or reduced training volume, whereas minor injuries were defined as small complaints for ≥ 2 days in a row. The included participants were comfortable with running on a treadmill with an average self‐reported comfort score of 7.74 ± 1.57 on a scale from 1 to 10. The study was approved by the local ethics committee (nr FHML‐REC/2023/017), was conducted according to the Declaration of Helsinki, and all participants provided written informed consent before testing.

### 2.3 Instruments

All experiments were performed on the computer‐assisted rehabilitation environment system (CAREN, Motek, The Netherlands) [25], which includes an instrumented split‐belt treadmill (belt length and width 2.15 × 0.5 m, 6.28‐kW motor per belt, 60 Hz belt speed update frequency, and 0–18 km∙h^−1^ speed range).

For respiratory gas analyses, participants wore a face mask (Hans Rudolp Inc., Shawnee, KS, USA) over the nose and mouth without detectable leakage. The mask was connected to a T‐piece that was placed in a free airstream (200 L min^−1^). Respiratory gases were captured using a previously validated indirect calorimeter (Omnical, Maastricht Instruments, Maastricht, The Netherlands) [18]. The system was calibrated automatically every 15–30 min using room air and a gas mixture of known composition. Lab temperature was kept constant at 21.6°C ± 0.9°C and relative humidity of 49.3% ± 4.8% during all test sessions.

### 2.4 Running Shoes

All participants were provided with four different running shoes (Table 1). These included two different brands of shoes with AFT (Asics MetaSpeed Sky+ [hereafter referred to as AFT1] and Kiprun KD900X [hereafter AFT2]), one traditional racing shoe (Saucony FastTwitch 9 [hereafter TRAD]), and a traditional neutral training shoe (Kiprun KS900 Light [hereafter NEUT]). The participants also brought their own regular training shoes (non‐AFT; hereafter OWN).

**TABLE 1:** Shoe characteristics.

	AFT1	AFT2	NEUT	TRAD
Mass (g)	193	211	252	171
Drop (mm)	5	4	8	5
Longitudinal bending stiffness (N∙mm^−1^)	10.5	9.5	4.3	5.7
Stack height (mm)	36.4	34.0	31.8	21.8
Peak force (N)	704	696	732	984
Max displacement (mm)	16.8	18.4	14.7	12.8
Energy input (J)	4.88	5.08	4.99	4.73
Energy return (J)	3.78	3.32	2.73	2.66
Energy loss (J)	1.10	1.76	2.26	2.07
Energy return (%)	78%	65%	56%	56%
Heel compression stiffness (N∙mm^−1^)	42.1	37.9	50.6	78.0

*Note:* AFT = shoe with Advanced Footwear Technology. Data in this table were provided by Decathlon, and were collected using procedures described in Morio et al. [26], with the exception of a mass of 8.1 kg being used instead of 6.5 kg, to mimic male impact forces. Bending stiffness was determined using a modified mechanical test from Healey et al. [27]. The supplemental file provides a brief overview of the tests done for assessing the mechanical shoe properties. Longitudinal bending stiffness refers to the resistance the shoe provides to bending around a mediolateral axis. Peak force reflects the peak vertical force during the impact test. The maximum displacement reflects the peak displacement of the shoe sole during the impact test. Energy input reflects the energy the shoe receives during an impact test (area under the force‐displacement curve during the loading phase of the impact test). Energy return reflects the energy the shoe returns after the impact (area under the force‐displacement curve during the unloading phase of the impact test), and the energy loss reflects the difference between the input and return energy. Heel compression stiffness refers to the resistance the heel provides to the loading phase of the impact test, calculated as the ratio between the peak force and the peak displacement.

### 2.5 Data Collection

Body mass was measured to the nearest 0.1 kg using a calibrated scale (Seca 799, Seca GmBh, Hamburg, Germany). The Achilles tendon moment arm was measured on the right leg in a standing position as the perpendicular distance from the center of the medial malleolus to the skin edge of the Achilles tendon. Foot length was measured as the straight line distance between the portion of the heel that touched the ground and the most anterior point of the second toe. Muscle architecture of the medial gastrocnemius and soleus from the right leg was assessed in a standing position using a 128‐element linear, flat‐shaped ultrasound transducer in B‐mode (Telemed ArtUs, Vilnius, Lithuania). Briefly, the probe was positioned over the mid‐belly and oriented in line with the fascicle plane as described previously [28]. Care was taken to minimize pressure as this could influence resting muscle architecture. Participants were also instructed to shift their center of pressure to the center of the foot during the measurements.

The participants then performed a standardized 8‐min warm‐up to familiarize themselves with treadmill running [29]. The warm‐up started at a speed of 2 m·s^−1^ (7.2 km∙h^−1^) and after 4 min was increased to the test speed of 2.78 m·s^−1^ (10 km∙h^−1^). After a brief rest period, the participants ran for ~5 min at 2.78 m·s^−1^ in each shoe following a randomized order. A duration of 5 min was used to ensure the final 1‐min steady‐state period could be used for biomechanical and gas exchange data analysis. Steady‐state gas exchange was also visually confirmed during the trials, with the duration of the trial being extended if a steady state was not reached. Three‐dimensional ground reaction forces, kinematics (not reported here), and gas exchange data were collected during all trials. Two of the five running shoes (AFT1 & NEUT) were worn twice to improve the accuracy of RE determination [19], resulting in a total of seven running trials per participant. It was considered unfeasible for participants to run in duplicate on all shoes because of the potential fatigue effects in this group of recreational runners. Participants were instructed to run as if they were running outside and to focus on the simulated virtual forest environment. The treadmill was kept level during all trials as this best matches overground physiology at speeds < 16 km·h^−1^ [30]. Rest between the different conditions was approximately 5 min.

After completing the first trial of each running shoe, the participants were asked to verbally rate the comfort of the shoe based on the standardized Running Footwear Comfort Assessment Tool (RUN‐CAT). This tool included assessment of overall comfort, heel cushioning, forefoot cushioning, medio‐lateral control, arch height, and heel cup fit [31]. Although the scale was originally developed as a visual analogue scale, we asked the runners to verbally rate each criterion from 0 to 10 to better mimic an in‐store experience where visual analogue scales are less likely to be used. For the shoes that were assessed twice, the first trial was used to assess comfort to match the comfort assessment for the other shoes.

### 2.6 Data Analysis

The rate of oxygen consumption (V̇O_2_) and carbon dioxide (V̇CO_2_) production were measured continuously and computed at 5‐s intervals throughout the running trials. To objectively confirm a steady rate of oxygen consumption, we fitted a linear regression over the analyzed 1‐min period (4–5 min). Although this analysis showed 10 slopes to increase significantly (*p* < 0.05; mean slope 1.17 ± 2.7 mL∙min^−1^), the magnitude was well below the recommended 150 mL∙min^−1^, which is often used to indicate a steady state of oxygen consumption [32]. Therefore, no data were excluded based on this analysis. Running trials with a respiratory exchange ratio (RER) > 1 were, however, excluded from RE analysis, resulting in the exclusion of complete data from three participants and two additional trials from other participants (thus yielding a total sample size of 38 participants). V̇O_2_ and V̇CO_2_ from the valid trials were subsequently used to determine substrate utilization using non‐protein equations [33], with energy cost being determined as the sum of fat and carbohydrate use. The energy cost was then expressed as kcal∙kg^−1^∙km^−1^.

The filtered kinetic data from the same last 1‐min were analyzed using custom‐written algorithms in Matlab (v 2019a, The MathWorks Inc., Natick, Massachusetts, USA) to compute the following spatiotemporal outcomes: ground contact time (ms), flight time (ms), stride time (ms), step frequency (steps∙min^−1^), and duty factor (%). Ground contact time represented the time between foot contact and toe‐off, flight time represented the time no foot contacted the ground, stride time represented the time from one foot contact to the next contact of the same foot, and step frequency was taken as the number of steps per minute. Duty factor was determined as the ratio between contact time and stride time and expressed as a percentage of stride time. Footstrike was identified when the vertical ground reaction force exceeded 20 N, and toe‐off was identified when the vertical ground reaction force dropped below 20 N. Results are only reported for the right leg, as preliminary analyses revealed similar results for the left leg. Using the last minute of a 5‐min run provides sufficient time for familiarization with new running shoes [34].

Ultrasound videos from each participant were exported to MATLAB to determine medial gastrocnemius and soleus fascicle length, and fascicle angle using a previously validated algorithm [35]. Within this algorithm, fascicle length was taken as the straight‐line distance between the two aponeuroses, and fascicle angle as the angle of the identified fascicle relative to the deep aponeurosis.

### 2.7 Statistical Analysis

For the primary aim, the estimated marginal means from a repeated measures linear mixed model fitted with a restricted maximum likelihood method (implemented in SPSS Version 25, IBM Corporation, Chicago, IL) were used to compare RE between the different shoes. To this purpose, the shoe was specified as a fixed effect, and a random intercept and slope were modeled per subject. Normality of the residuals was inspected visually using Q‐Q plots and histograms. A similar model was used to compare spatiotemporal running metrics and comfort outcomes between the shoes. Pairwise comparisons were used to compare all outcomes between the shoes. To correct for the family‐wise error rate with multiple comparisons, the threshold for statistical significance was set at 0.05/4 = 0.0125. We also computed Pearson correlations between shoe characteristics (Table 1) and RE. Correlations were interpreted as < 0.1 trivial; 0.1–0.29 small; 0.30–0.49 moderate; 0.5–0.69 large; 0.7–0.89 very large; 0.9–0.99 nearly perfect [36].

For the secondary aim, we first explored the variation in shoe ranking across individuals. To this purpose, Kendall's Coefficient of Concordance was used to compare the ranking of shoes in RE across individuals. This test, for example, provides information on which AFT shoe 1 is always the shoe in which individuals are most economical, and by doing so, it provides information on the potential individual variation in response to different shoes.

To investigate whether biomechanical, anthropometric, muscle architectural, or comfort outcomes mediate the effect of shoes on RE, we first computed delta (Δ) scores by subtracting the RE, spatiotemporal, anthropometric, muscle architectural, or comfort data from each shoe from the values of a reference shoe. The traditional neutral running shoe was chosen as a reference shoe because it was a traditional neutral training shoe and was also measured twice. For shoes that were measured twice, we first computed an average across both measurements before further analyses. Pearson correlation coefficients were then calculated between ΔRE and the Δ‐value of a spatiotemporal, anthropometric, muscle architecture, or comfort outcome. Males and females were combined for all analyses because previous studies did not show differences in responses to footwear between sexes [10, 37], and because a relatively small number of females participated in the present study. The participants' own shoes were not used in this analysis because of the diversity in the shoes used.

## 3 Results

### 3.1 Group Mean Effects

**FIGURE 1:**
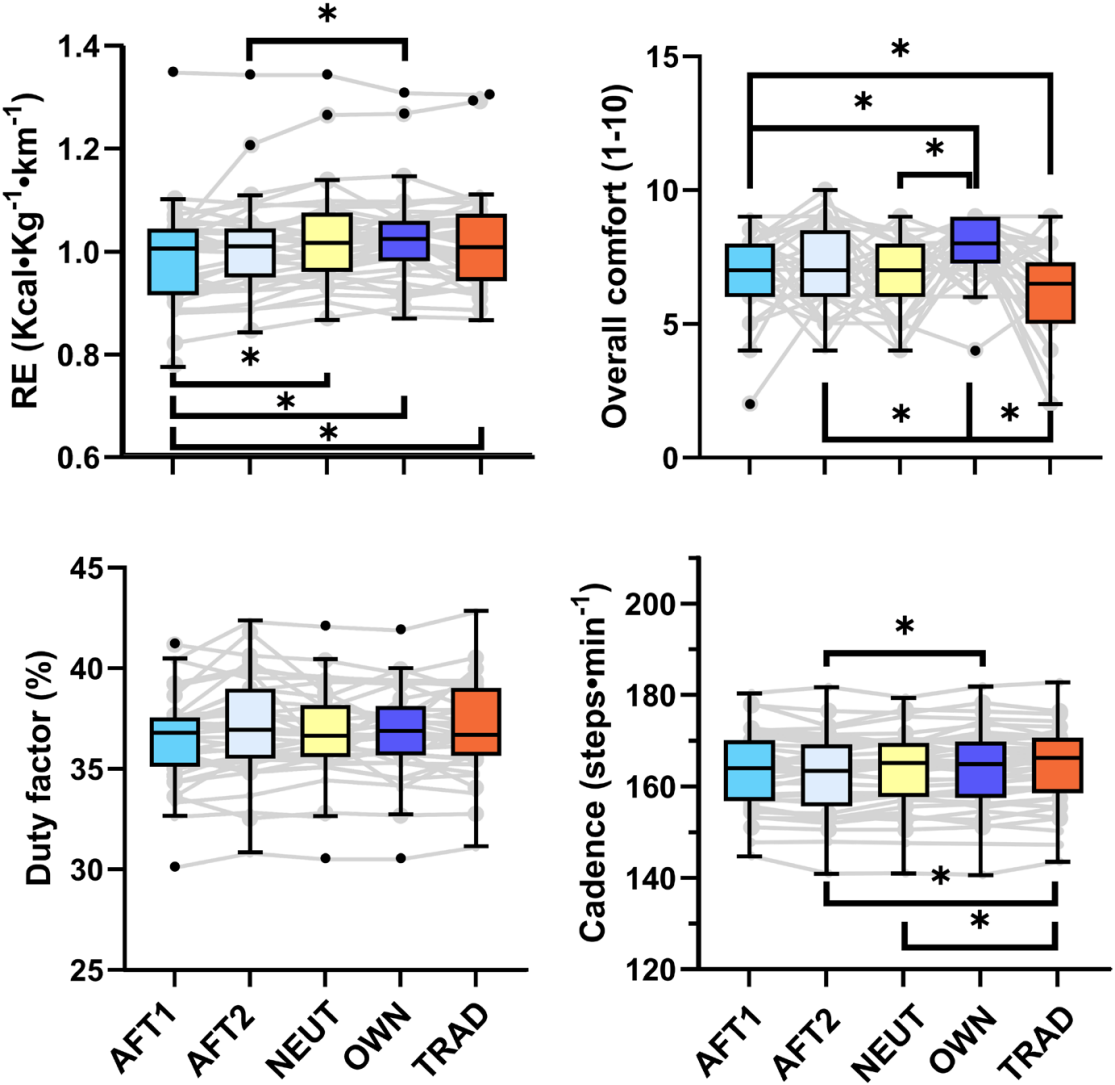
Running economy (left top), overall comfort (rigtht top), duty factor (left bottom), and Cadence (right bottom) for the five different shoes. Boxplots depict the median and interquartile range, Tukey whiskers, with dots representing outliers (> 1.5 times the interquartile range). Gray lines and dots depict individual data points. Statistically significant (*p* < 0.0125) differences are indicated by an asterisk (*).

Running economy, spatiotemporal outcomes, and comfort for each shoe are provided in Table 2 and depicted in Figure 1A. Table 3 provides a comparison of RE between each shoe. Briefly, RE was significantly improved by 2%–4% in AFT1 compared to all shoes, with the difference relative to the AFT2 being non‐significant (Table 3, Figure 1A). The difference in spatiotemporal metrics and comfort outcomes is partly presented in Figure 1 and further detailed in Supplemental file Table S1–S10. Table S11 provides the correlation coefficients between shoe characteristics and RE, and shoe characteristics and overall comfort, as well as selected other comfort outcomes. Mean ± SD foot length and Achilles tendon moment arms were 26.9 ± 1.71 cm and 5.81 ± 0.72 cm, respectively. Mean ± SD fascicle length and fascicle angle were 41.3 ± 9.32 mm and 21.1° ± 4.44°, and 33.1 ± 11.3 mm and 20.6° ± 6.32° for the gastrocnemius medialis and soleus, respectively.

**TABLE 2:** Mean ± SD running economy and selected spatiotemporal metrics per shoe.

	AFT1	AFT2	NEUT	TRAD	OWN
Energy cost outcomes					
Energy cost (kcal∙kg^−1^∙km^−1^)	0.99 ± 0.10	1.01 ± 0.09	1.03 ± 0.10	1.02 ± 0.10	1.03 ± 0.09
Spatiotemporal outcomes					
Contact time (ms)	269 ± 21	274 ± 22	271 ± 21	271 ± 21	271 ± 23
Flight time (ms)	100 ± 19	96 ± 22	98 ± 19	95 ± 19	97 ± 19
Duty factor (%)	37 ± 2	37 ± 2	37 ± 2	37 ± 2	37 ± 2
Cadence (steps∙min^−1^)	163 ± 8	163 ± 9	163 ± 9	164 ± 9	164 ± 9
Comfort outcomes					
Overall comfort	6.9 ± 1.5	7.1 ± 1.5	6.9 ± 1.3	6.1 ± 1.9	7.9 ± 1.1
Heel cushioning comfort	6.7 ± 1.7	7.1 ± 1.8	6.8 ± 1.6	5.5 ± 1.7	7.4 ± 1.4
Forefoot cushioning comfort	6.9 ± 1.6	7.1 ± 1.7	6.1 ± 1.7	5.4 ± 2.0	7.0 ± 1.7
Medio‐lateral control	6.1 ± 1.9	6.0 ± 1.8	6.9 ± 1.9	6.6 ± 1.9	8.1 ± 1.5
Arch height comfort	6.3 ± 2.0	6.6 ± 1.9	6.6 ± 1.6	5.8 ± 2.1	7.4 ± 1.9
Heel cup fit	6.4 ± 2.0	6.6 ± 1.7	7.2 ± 1.3	6.5 ± 1.5	7.9 ± 1.1

**TABLE 3:** Pairwise comparisons of RE between the five shoe models.

Compared shoe	Reference shoe	Marginal mean ± SE (kcal∙kg^−1^∙km^−1^)	Mean ± SD % difference in RE	*p*
AFT1	AFT2	−0.02 ± 0.07	−2.14 ± 3.93	0.03
	NEUT	−0.03 ± 0.07	−3.99 ± 5.03	0.001*
	OWN	−0.03 ± 0.07	−4.01 ± 4.99	0.001*
	TRAD	−0.02 ± 0.07	−3.04 ± 6.19	0.001*
AFT2	NEUT	−0.02 ± 0.07	−1.82 ± 2.65	0.02
	OWN	−0.02 ± 0.07	−1.83 ± 3.19	0.01*
	TRAD	−0.01 ± 0.07	−0.88 ± 3.99	0.21
NEUT	OWN	−0.01 ± 0.07	−0.01 ± 2.76	0.84
	TRAD	0.01 ± 0.07	0.92 ± 3.28	0.24
OWN	TRAD	0.01 ± 0.07	0.93 ± 3.53	0.17

*Note:* Statistically significant differences (*p* < 0.0125) after decreasing the threshold value for statistical significance are indicated by asterisks (*). Negative numbers indicate a lower energy cost (i.e., improved RE) in the compared shoe versus the reference shoe.

### 3.2 Variability in Shoe Rank Order

Kendall's Coefficient of Concordance was 0.16, *p* = 0.001, thus indicating low concordance in the ranking of the shoe order across the different participants. In other words, there was substantial variability in the rank order of which shoe was most economical across individuals.

### 3.3 Mediating Effect of Anthropometrical, Muscle Architectural, Spatiotemporal, and Comfort Metrics on RE

**FIGURE 2:**
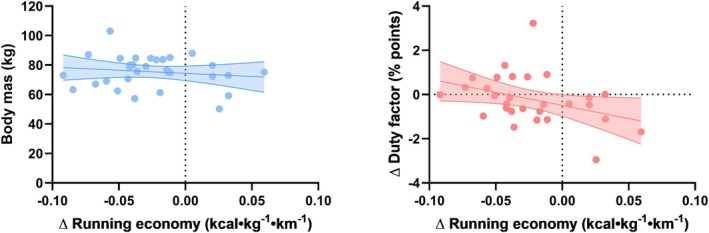
Scatterplots depicting the correlation between (left) body mass and the change in running economy between the AFT1 and neutral reference shoe, and (right) the change in duty factor and change in running economy between the AFT1 and neutral reference shoe. Negative running economy values depict a lower energy cost in the AFT1 shoe, while positive duty factor scores depict a higher duty factor in the AFT1 shoe (i.e., more time spent on the ground relative to stride time). Dots depict individual datapoints, and the regression line with shaded area depicts the linear regression line with 95% confidence bands.

Table 4 shows the correlations between anthropometrical, muscle architectural, relative spatiotemporal, relative comfort metrics, and relative RE for the three shoes (relative to the reference shoe). Figure 2 also depicts selected relationships. Although several correlations were quantitatively potentially relevant, only three correlations were statistically significant after correcting for multiple comparisons (GM fascicle length and contact time in AFT2 shoe, and arch height comfort in the traditional racing flat).

**TABLE 4:** Mean (95% confidence intervals) correlation coefficients between anthropometrical, muscle architectural, spatiotemporal, and comfort metrics and relative RE.

Outcome	AFT1	AFT2	TRAD	Average
Anthropometric and muscle architecture outcomes				
Foot length	−0.10 (−0.44 to 0.26)	0.02 (−0.37 to 0.40)	0.07 (−0.28 to 0.40)	0.00 (−0.36 to 0.35)
Achilles tendon moment arm	0.06 (−0.33 to 0.43)	0.29 (−0.13 to 0.62)	−0.33 (−0.62 to 0.03)	0.01 (−0.36 to 0.36)
Body mass	−0.14 (−0.47 to 0.23)	0.39 (0.01 to 0.67)	−0.23 (−0.58 to 0.19)	0.01 (−0.35 to 0.36)
GM fascicle length	−0.09 (−0.58 to 0.44)	−0.74 (−0.92 to −0.20)*	0.08 (−0.43 to 0.55)	−0.25 (−0.64 to 0.26)
GM fascicle angle	−0.22 (−0.66 to 0.33)	0.41 (−0.21 to 0.80)	0.13 (−0.39 to 0.59)	0.11 (−0.42 to 0.57)
SOL fascicle length	−0.23 (−0.66 to 0.32)	−0.25 (−0.74 to 0.41)	0.15 (−0.46 to 0.67)	−0.11 (−0.62 to 0.47)
SOL fascicle angle	0.35 (−0.20 to 0.73)	−0.02 (−0.61 to 0.59)	−0.16 (−0.67 to 0.46)	0.06 (−0.49 to 0.59)
Spatiotemporal outcomes				
Contact time	−0.29 (−0.60 to 0.09)	−0.48 (−0.75 to −0.07)*	−0.30 (−0.66 to 0.16)	−0.36 (−0.67 to 0.06)
Flight time	0.36 (−0.01 to 0.64)	0.24 (−0.20 to 0.60)	0.23 (−0.55 to 0.14)	0.28 (−0.25 to 0.46)
Duty factor	−0.38 (−0.66 to −0.02)	−0.39 (−0.70 to 0.04)	−0.28 (−0.58 to 0.08)	−0.35 (−0.65 to 0.04)
Cadence	−0.16 (−0.50 to 0.22)	0.22 (−0.22 to 0.59)	0.02 (−0.37 to 0.34)	0.03 (−0.36 to 0.38)
Comfort outcomes				
Overall comfort	0.48 (0.73 to 0.29)	−0.03 (−0.48 to 0.43)	−0.02 (−0.39 to 0.36)	0.14 (−0.05 to 0.36)
Heel cushioning comfort	0.44 (0.06 to 0.71)	−0.02 (−0.44 to 0.41)	−0.14 (−0.49 to 0.25)	0.10 (−0.29 to 0.46)
Forefoot cushioning comfort	0.22 (−0.19 to 0.57)	−0.26 (−0.61 to 0.17)	−0.11 (−0.46 to 0.27)	−0.05 (−0.42 to 0.34)
Medio‐lateral control	0.23 (−0.20 to 0.59)	−0.04 (−0.43 to 0.36)	0.22 (−0.18 to 0.56)	0.14 (−0.27 to 0.50)
Arch height comfort	0.34 (−0.10 to 0.67)	−0.01 (−0.47 to 0.46)	−0.65 (−0.84 to −0.31)*	−0.11 (−0.47 to 0.48)
Heel cup fit	0.43 (0.07 to 0.69)	0.01 (−0.39 to 0.41)	−0.14 (−0.51 to 0.28)	0.10 (−0.28 to 0.46)

Abbreviations: GM = gastrocnemius medialis, SOL = soleus.

* Indicates a significant correlation after adjusting for multiple comparisons (*p* < 0.0125).

## 4 Discussion

The aims of this study were to (1) investigate the effects of two shoes with AFT on RE at speeds representative of recreational runners relative to a traditional neutral shoe, traditional racing flat, and the participant's own shoes, and (2) investigate whether running biomechanics, anthropometrics, muscle architecture, or comfort mediate the effect of shoes on RE. Overall, our findings show that AFT can enhance RE also at speeds typical for recreational runners, although the magnitude of the effect differed (albeit non‐significantly) between the two investigated brands. Further, individual variability in RE within different shoes does not seem consistently strongly mediated by anthropometrics or muscle architecture, or spatiotemporal metrics, although some spatiotemporal metrics showed consistent non‐significant correlations of at least small magnitude that may be worthwhile to explore in further research.

### 4.1 Effect of Different Shoes on RE

Running economy in the AFT1 shoe was significantly improved by 2%–4% compared to all other shoes, except for AFT2, where there was a non‐significant difference of 2% (Figure 1). Similarly, AFT2 quantitatively improved RE compared to the other shoes by 1%–2%. These findings were partially expected as both AFT shoes incorporate features such as a highly compliant and resilient midsole, a carbon plate, and a curved sole geometry. Previous studies have shown that such features can improve RE compared to traditional racing shoes and regular training shoes, although the exact mechanisms for the performance‐enhancing effect of shoes with AFT remain debated [37]. For example, some studies show a beneficial effect of a moderately stiff carbon plate [38], yet others suggest no additional effect of the carbon plate within AFT shoes [27, 39]. Similarly, the compliance and resilience (energy return) of the midsole have been suggested to play an important role in the performance‐enhancing effect in some [40], but not all studies [39]. Notably, the benefit of the energy returned by compliant midsoles for enhancing RE has recently also been debated [41]. The conflicting findings are at least partly related to differences between loading in bench‐top experiments and actual running [42] and likely also result from the complex interaction between diverse shoe features.

In an attempt to gain further insight into the potential mechanisms for the differential effects of the investigated shoes on RE in the present study, we explored the relationship between selected shoe characteristics and RE at a group level (Table S11). This analysis showed that the energy loss was most strongly associated with RE (*r* = 0.99), such that lower energy loss (i.e., less energy dissipated) was associated with better RE. It has previously been shown that the midsole foam properties have an important contribution to the energy return [40], and differences in these properties between the shoes in the present study therefore have an important effect on RE. Notably, previous studies typically quantified the energy return value. However, part of the larger energy return when compressing shoes is used to return the compressed material to its original state, and energy loss has therefore been suggested to be more relevant in the context of RE [41]. Further, an increased longitudinal bending stiffness was also very strongly associated with better RE (*r* = −0.92). Interestingly, the relationship also appeared to be linear, with no maximum optimal stiffness. This is in contrast to previous research that showed an intermediate stiffness value to be optimal for enhancing RE [38]. This apparent conflict may be because all longitudinal bending stiffness values in the present study (4–10 N∙mm^−1^) were lower than the optimal stiffness in previous research (35 N∙mm^−1^) [38], although this difference may also at least partly reflect different methods to assess longitudinal bending stiffness. Increasing the mass of the shoe increases the energy cost of running by ~1% for every 100 g added [43]. We also observed a moderate correlation (*r* = 0.46) between shoe mass and RE in line with these findings. Notably, as this correlation was weaker than the correlation between energy return and RE, increasing the energy return or bending stiffness is more important than decreasing the mass of the shoe for enhancing RE, at least within the range of masses (171–252 g) and energy return values (56%–78%) investigated here. Indeed, the traditional racing shoe had the lowest mass in the present study, but RE was worse compared to two shoes that had a slightly heavier mass, but also had better energy return (Table 1). Similar findings have been observed previously [9, 44]. Although our analysis also showed a moderate magnitude correlation between higher stack height and lower energy cost, increasing the midsole thickness in isolation does not improve RE [45] or even reduce RE [40], thus this effect likely reflects the different material properties associated with thicker insoles (i.e., more compliant and resilient material [40]), rather than stack height/midsole thickness per se. In this context, it is also interesting to hypothesize on the potential reason for the ~2% difference in RE between the AFT shoes. First, assuming the energy cost of running increases by ~1% for every 100 g added [43], the 18 g difference in mass would explain only 0.18% of the difference in RE between shoes, although the exact magnitude also depends on running speed [46]. The remaining difference may therefore primarily reflect a greater longitudinal bending stiffness in the AFT1 shoe, which can enhance RE by reducing the calf muscle shortening velocity and shortening distance [47].

Overall comfort also differed significantly between the different shoes, with the participant's own shoe being rated as most comfortable, in line with previous research [4]. This is likely because runners partly select their shoes based on comfort [48], and because repetitive running in the shoes further increases shoe fit to the specific foot, thereby potentially increasing comfort. The traditional racing shoe was rated as least comfortable overall, likely because of its thin midsole and resulting lower shock absorption, in particular at the forefoot (Table 2, Table S7). In line with previous studies showing lower frontal plane ankle stability with higher midsole thickness [45], perceived medio‐lateral balance was lowest in shoes with AFT, likely because of the thick midsole (Table 2, Table S8). In further support of this, we also observed a moderate negative relationship (*r* = −0.53) between stack height and perceived medio‐lateral stability across the different shoes (Table S11). Similarly, the runners may also have been able to perceive the heel compression stiffness differences, as indicated by a moderate correlation (*r* = 0.63) between perceived heel cushioning and heel compression stiffness from mechanical testing (Table S11).

### 4.2 Spatiotemporal, Anthropometrical, Muscle Architectural, or Comfort Metrics as Mediating Factors of RE

Although there were significant differences in RE between the investigated shoes at a group level, there was also substantial variability in the ranking of the most economical shoe across individuals (Kendall's Coefficient of Concordance 0.16, *p* = 0.001). Although this undoubtedly partly reflects measurement error in RE, a part of this variation also reflects individual variation in the response to different shoes. Such a finding reinforces previous suggestions that shoe wear needs to be individualized. It has previously been suggested that the response to different shoes is partly mediated by running biomechanics [20]. Based on this potential mediating effect of running biomechanics on RE across different shoes, we explored whether changes in spatiotemporal metrics were associated with changes in RE for each shoe. A similar analysis was done for anthropometrics, muscle architecture, and comfort.

Anthropometrical outcomes such as body mass, foot length, and Achilles tendon moment arm, or muscle architectural outcomes, did not (consistently) mediate the effect of shoes on RE across the three different shoes investigated (Table 4). Although these varying correlation magnitudes and directions could suggest that the mediating effect differs between shoes by its interaction with specific shoe features, it could also suggest that the anthropometrics or muscle architectural characteristics investigated in the present study do not simply mediate the effect of shoes on RE, with the small correlations observed within some shoes reflecting measurement error and variability. In support of the latter interpretation, most previous studies also reported no relationship between body mass and relative RE across different shoes [10, 23, 49, 50]. Further, re‐analyses of the data provided by Beck and colleagues [49] showed also only trivial and non‐significant correlations between foot length and relative RE (average *r* = −0.03), or Achilles tendon moment arm and relative RE (average *r* = 0.00) when comparing RE in the shoes with three longitudinal stiffness values to the most compliant shoe.

Spatiotemporal running metrics generally showed quantitatively stronger magnitude associations with relative RE than the anthropometrical outcomes, although none of the associations were statistically significant. It is, however, interesting to note that in all three shoes investigated, a relatively longer contact time, a relatively shorter flight time, and a resulting higher relative proportion of stride time spent on the ground (i.e., higher duty factor) were weakly to moderately yet non‐significantly associated with a better relative RE (Table 4). In other words, individuals who prolonged their ground contact time more in the shoes with AFT, or in the traditional racing shoe, tended to show a relatively larger benefit in RE in those shoes as compared to the reference shoe, possibly because of a slower fascicle shortening velocity with longer contact times [21, 51]. Nevertheless, as the correlations were non‐significant, further research is required to substantiate this notion. Further, the reason why some individuals may change their spatiotemporal metrics more than others in response to different footwear requires further research. Cadence was not associated with relative RE (also a trivial magnitude correlation). Previous studies typically also observed no significant correlations between relative spatiotemporal metrics and relative RE. Beck and co‐workers [49] for example, found no significant relationship (*p* = 0.135) between the change in contact time and the change in RE between a shoe with and without a carbon plate. Re‐analyses of the data provided by the authors showed similar non‐significant, trivial to small correlations between relative contact time and relative RE in all three shoes (average *r* = −0.01), and similar non‐significant and trivial to small correlations between relative step frequency and relative RE (average *r* = 0.03). Similarly, Hoogkamer and colleagues [6] found no significant association between relative contact time or step frequency and relative RE in three different shoes. Re‐analysis of the data provided by the authors indeed showed that relative contact time was not consistently correlated with relative RE in the Nike Vaporfly prototype or Adidas shoe vs. the traditional racing shoe (average *r* = 0.07 across the two shoes and three speeds). In a multiple regression analysis, the percent changes in peak vertical ground reaction force, step frequency, and contact time, however, explained 20% of the variance in the reductions in energetic cost between the Nike Vaporfly and traditional racing shoe, with longer contact times being associated with better relative RE, similar to the non‐significant association in our study. Finally, Barnes and Kilding [9] also, mostly found trivial to small correlations between relative RE and relative spatiotemporal metrics between the Nike Vaporfly and Adidas shoes across all speeds. However, when contact time, stride length, and stride frequency were combined in a regression analysis, they contributed 0.17% and 0.95% to the 2.60% and 4.20% improvement in RE, respectively. Overall, when these findings are combined with those of the present study, they suggest that changes in spatiotemporal metrics in isolation may be at best weakly associated with changes in RE.

A recent systematic review found increased comfort to be associated with better RE [24]. While one significant correlation was observed between relative RE and relative comfort outcomes in the present study (Table 4), the correlations were highly variable between the different shoes, and overall trivial to small when averaged across the three shoes (Table 4). Therefore, our findings do not provide strong support for the notion that those who experience specific shoes as more comfortable are also more economical in those shoes. The apparently conflicting findings with the systematic review may be because the studies compared group‐level comfort and RE, and because studies included in the systematic review could use barefoot running and foot orthoses, which may have more substantial effects on comfort and RE than a comparison between different ‘regular’ running shoes. Moreover, four of the six included studies also controlled for mass differences between the shoes, whereas our study did not correct for this to enhance ecological validity.

### 4.3 Limitations

This study has several strengths, but also limitations that should be kept in mind when interpreting the findings. Strengths include the relatively large and mixed‐sex sample, the use of five running shoes with relatively widely varying characteristics, and the integration of biomechanical, anthropometrical, muscle architectural, and perceptual (comfort) measurements as possible mediating factors. A first limitation relates to the use of single trials for assessing RE in some shoes. Specifically, while multiple measurements of RE per shoe are recommended to reduce measurement error [19], we assessed RE in duplicate for only two of the five shoes. This decision was made to reduce the influence of fatigue in this group of recreational runners, where multiple trials could otherwise have offset the benefit of having more measurements. Nevertheless, the single trials for some shoes could have increased measurement error and thereby reduced our ability to establish relationships between relative RE and relative anthropometric, spatiotemporal, or comfort outcomes. However, the gas exchange system used in the present study is among the most accurate and reliable systems available [18], thus reducing the error associated with a single measurement. Moreover, we used a relatively large sample size to further reduce the impact of measurement errors on the investigated relationships. Finally, studies that used multiple measurements for all shoes (e.g., Hoogkamer et al. [6]) also found that changes in body mass or spatiotemporal metrics across shoes could not accurately explain changes in RE across shoes, thus further increasing our confidence in the observed findings. A second limitation relates to our assessment of variability in the rank order of RE across shoes. Specifically, we ranked running shoes from 1 to 5 according to their relative RE per individual, but the differences between shoes were sometimes very small. The Kendall's Coefficient of Concordance may therefore overestimate the actual variability in response to different shoes, whereby some shoes may be considered equally economical, yet still ranked in a specific order. A third and final consideration is that the participants were asked to verbally rate comfort on a 0–10 scale, rather than by using a visual analogue scale. While the use of a visual analogue scale may offer increased resolution, we purposely used a verbal assessment to increase ecological validity towards an in‐store shoe purchase where sales persons would likely also ask the runner to verbally rate the comfort, instead of using a scale. Similarly, it has been shown that subjective comfort ratings are more reliable when the assessment is repeated [31], but we chose not to perform repeated runs to better mimic an in‐store experience.

### 4.4 Perspective

There are several implications from this study. First, our findings add to a growing body of literature that shows which include AFT features (Asics MetaSpeed Sky and KiprunKD 900X) can enhance RE compared to traditional racing shoes (Saucony Fastwitch 9), or training shoes (Kiprun KS900 Light, and the participants own shoes) also at speeds representative of recreational runners. Notably, the enhancement of RE by the Asics shoe (4%) was in line with previous studies showing a similar enhancement at higher running speeds (2.8%–4.4% at 14–18 km∙h^−1^) [6, 8, 9]. Using established relationships between a given improvement in RE and a given improvement in velocity [52], it can be determined that the 2%–4% benefit in RE with the Asics or KiprunKD shoes, respectively, would improve marathon time by 5.5–9.5 min (2.2%–4.4%), respectively, for a 4:13:00 marathon runner. Such improvements are arguably of relevant magnitude.

Second, our findings indicate that easy‐to‐obtain anthropometrical characteristics such as foot length, Achilles tendon moment arm, or body mass do not mediate the effect of shoes on RE, and such outcomes can therefore not be used to estimate if a given individual is likely to benefit more from a specific shoe. Similarly, a change in perceived comfort between two shoes is unlikely to reflect the change in running economy between two shoes and can therefore not be used to recommend which shoe is most economical. Finally, changes in selected spatiotemporal metrics such as contact time, flight time, and duty factor also showed no significant relationships with the change in RE, but the small to moderate magnitude for some outcomes may be of potential relevance and requires future research using a larger sample size.

## 5 Conclusion

Advanced footwear technology enhanced RE at speeds typical for recreational runners, although with variable magnitude (2%–4%) across the two different brands. Further, neither anthropometrics, muscle architecture, spatiotemporal metrics, nor comfort strongly or consistently influenced the effect of shoes on RE.

## Author Contributions

B.V.H. conceived the study, S.P. and B.V.H. collected the data, R.C., S.P., Z.B., and B.V.H. analyzed the data, B.V.H. wrote the first draft of the manuscript, and R.C., K.M. provided comments and edits. All authors approved the final version.

## Conflicts of Interest

The authors declare no conflicts of interest.

## Supporting information


Table S1.

## Data Availability

The data that support the findings of this study are available from the corresponding author upon reasonable request.

## References

[1] Statista , “Number of Running and Jogging Participants in the United States From 2010 to 2023,” 2023, accessed January 22, 2025, https://www.statista.com/statistics/190303/running‐participants‐in‐the‐us‐since‐2006/.

[2] V. Rodrigo‐Carranza , F. González‐Mohíno , J. Santos‐Concejero , and J. M. González‐Ravé , “Impact of Advanced Footwear Technology on Elite Men's in the Evolution of Road Race Performance,” Journal of Sports Sciences 40, no. 23 (2022): 2661–2668. 36814065 10.1080/02640414.2023.2183103

[3] V. Rodrigo‐Carranza , F. González‐Mohíno , J. Santos del Cerro , J. Santos‐Concejero , and J. M. González‐Ravé , “Influence of Advanced Shoe Technology on the Top 100 Annual Performances in Men's Marathon From 2015 to 2019,” Scientific Reports 11, no. 1 (2021): 22458. 34789828 10.1038/s41598-021-01807-0 PMC8599511

[4] K. Hébert‐Losier , S. J. Finlayson , M. W. Driller , B. Dubois , J. F. Esculier , and C. M. Beaven , “Metabolic and Performance Responses of Male Runners Wearing 3 Types of Footwear: Nike Vaporfly 4%, Saucony Endorphin Racing Flats, and Their Own Shoes,” Journal of Sport and Health Science 11, no. 3 (2022): 275–284. 33264686 10.1016/j.jshs.2020.11.012 PMC9189709

[5] J. H. Hudgins , J. T. Pastina , I. L. Gillis , et al., “The Ability of Stryd Footpod Metrics to Reflect Changes in Metabolic Power Between Running Shoe Types,” Journal of Sports Sciences 42, no. 23 (2024): 2229–2241. 39565291 10.1080/02640414.2024.2426903

[6] W. Hoogkamer , S. Kipp , J. H. Frank , E. M. Farina , G. Luo , and R. Kram , “A Comparison of the Energetic Cost of Running in Marathon Racing Shoes,” Sports Medicine 48, no. 4 (2018): 1009–1019, 10.1007/s40279-017-0811-2. 29143929 PMC5856879

[7] E. Martinez, 3rd , W. Hoogkamer , D. W. Powell , et al., “The Influence of “Super‐Shoes” and Foot Strike Pattern on Metabolic Cost and Joint Mechanics in Competitive Female Runners,” Medicine and Science in Sports and Exercise 56 (2024): 1337–1344. 38376997 10.1249/MSS.0000000000003411

[8] I. Hunter , A. McLeod , D. Valentine , T. Low , J. Ward , and R. Hager , “Running Economy, Mechanics, and Marathon Racing Shoes,” Journal of Sports Sciences 37, no. 20 (2019): 2367–2373. 31223054 10.1080/02640414.2019.1633837

[9] K. R. Barnes and A. E. Kilding , “A Randomized Crossover Study Investigating the Running Economy of Highly‐Trained Male and Female Distance Runners in Marathon Racing Shoes Versus Track Spikes,” Sports Medicine 49, no. 2 (2019): 331–342, 10.1007/s40279-018-1012-3. 30374945

[10] D. P. Joubert , T. A. Dominy , and G. T. Burns , “Effects of Highly Cushioned and Resilient Racing Shoes on Running Economy at Slower Running Speeds,” International Journal of Sports Physiology and Performance 18, no. 2 (2023): 164–170. 36626911 10.1123/ijspp.2022-0227

[11] A. Werkhausen , M. Lund‐Hansen , L. Wiedenbruch , K. Peikenkamp , and H. Rice , “Technologically Advanced Running Shoes Reduce Oxygen Cost and Cumulative Tibial Loading Per Kilometer in Recreational Female and Male Runners,” Scientific Reports 14, no. 1 (2024): 11903. 38789519 10.1038/s41598-024-62263-0 PMC11126714

[12] G. P. Paradisis , E. Zacharogiannis , A. Bissas , and B. Hanley , “Recreational Runners Gain Physiological and Biomechanical Benefits From Super Shoes at Marathon Paces,” International Journal of Sports Physiology and Performance 18, no. 12 (2023): 1420–1426. 37734742 10.1123/ijspp.2023-0115

[13] M. Knopp , B. Muniz‐Pardos , H. Wackerhage , et al., “Variability in Running Economy of Kenyan World‐Class and European Amateur Male Runners With Advanced Footwear Running Technology: Experimental and Meta‐Analysis Results,” Sports Medicine 53, no. 6 (2023): 1255–1271. 36862339 10.1007/s40279-023-01816-1 PMC10185608

[14] C. Heyde , A. Nielsen , K. Roecker , et al., “The Percentage of Recreational Runners That Might Benefit From New Running Shoes. A Likely Scenario,” Footwear Science 14, no. 3 (2022): 163–172.

[15] F. Horst , F. Hoitz , D. Slijepcevic , et al., “Identification of Subject‐Specific Responses to Footwear During Running,” Scientific Reports 13, no. 1 (2023): 11284. 37438380 10.1038/s41598-023-38090-0 PMC10338529

[16] J. Koegel , S. Huerta , M. Gambietz , et al., “Clustering Runners' Response to Different Midsole Stack Heights: A Field Study,” Sensors 24, no. 14 (2024): 4694. 39066091 10.3390/s24144694 PMC11280980

[17] M. Chollet , S. Michelet , N. Horvais , S. Pavailler , and M. Giandolini , “Individual Physiological Responses to Changes in Shoe Bending Stiffness: A Cluster Analysis Study on 96 Runners,” European Journal of Applied Physiology 123, no. 1 (2023): 169–177. 36229743 10.1007/s00421-022-05060-9

[18] B. Van Hooren , T. Souren , and B. C. Bongers , “Accuracy of Respiratory Gas Variables, Substrate, and Energy Use From 15 CPET Systems During Simulated and Human Exercise,” Scandinavian Journal of Medicine & Science in Sports 34, no. 1 (2024): e14490, 10.1111/sms.14490. 37697640

[19] Z. B. Barrons , V. Rodrigo‐Carranza , M. Bertschy , and W. Hoogkamer , “The Fallacy of Single Trials: The Need for Multiple Trials in Assessing Running Economy Responses in Advanced Footwear Technology,” Sports Medicine 54 (2024): 1–4. 10.1007/s40279-023-01991-1 38407747

[20] M. J. Connick and G. A. Lichtwark , “Individualization of Footwear for Optimizing Running Economy: A Theoretical Framework,” Journal of Applied Biomechanics 1 (2024): 1–7. 10.1123/jab.2024-0109 39662312

[21] B. Van Hooren , I. Jukic , M. Cox , et al., “The Relationship Between Running Biomechanics and Running Economy: A Systematic Review and Meta‐Analysis of Observational Studies,” Sports Medicine 54 (2024): 1269–1316. 38446400 10.1007/s40279-024-01997-3 PMC11127892

[22] K. Z. Takahashi , M. T. Gross , H. Van Werkhoven , et al., “Adding Stiffness to the Foot Modulates Soleus Force‐Velocity Behaviour During Human Walking,” Scientific Reports 6, no. 1 (2016): 29870. 27417976 10.1038/srep29870 PMC4945910

[23] N. Flores , G. Rao , E. Berton , and N. Delattre , “Increasing the Longitudinal Bending Stiffness of Runners' Habitual Shoes: An Appropriate Choice for Improving Running Performance?,” Proceedings of the Institution of Mechanical Engineers, Part P: Journal of Sports Engineering and Technology 237, no. 3 (2023): 121–133.

[24] K. Van Der Alsenoy , M. Linden , O. Girard , M. L. van der Linden , and D. Santos , “Increased Footwear Comfort Is Associated With Improved Running Economy–A Systematic Review and Meta‐Analysis,” European Journal of Sport Science 23, no. 1 (2023): 121–133. 34726119 10.1080/17461391.2021.1998642

[25] A. J. van den Bogert , T. Geijtenbeek , O. Even‐Zohar , F. Steenbrink , and E. C. Hardin , “A Real‐Time System for Biomechanical Analysis of Human Movement and Muscle Function,” Medical & Biological Engineering & Computing 51, no. 10 (2013): 1069–1077, 10.1007/s11517-013-1076-z. 23884905 PMC3751375

[26] C. Y. Morio , L. Bouten , S. Duraffourg , and N. Delattre , “A Multidisciplinary Approach to the Engineering of Footwear Cushioning: A Practical Example of Gym Training Shoes,” Proceedings of the Institution of Mechanical Engineers, Part P: Journal of Sports Engineering and Technology 236, no. 1 (2022): 5–16.

[27] L. A. Healey and W. Hoogkamer , “Longitudinal Bending Stiffness Does Not Affect Running Economy in Nike Vaporfly Shoes,” Journal of Sport and Health Science 11, no. 3 (2022): 285–292. 34280602 10.1016/j.jshs.2021.07.002 PMC9189697

[28] B. Bolsterlee , S. C. Gandevia , and R. D. Herbert , “Ultrasound Imaging of the Human Medial Gastrocnemius Muscle: How to Orient the Transducer so That Muscle Fascicles Lie in the Image Plane,” Journal of Biomechanics 49 (2016): 1002–1008, 10.1016/j.jbiomech.2016.02.014. 26905734

[29] B. Van Hooren , J. T. Fuller , J. D. Buckley , et al., “Is Motorized Treadmill Running Biomechanically Comparable to Overground Running? A Systematic Review and Meta‐Analysis of Cross‐Over Studies,” Sports Medicine 50, no. 4 (2020): 785–813, 10.1007/s40279-019-01237-z. 31802395 PMC7069922

[30] J. R. Miller , B. Van Hooren , C. Bishop , et al., “A Systematic Review and Meta‐Analysis of Crossover Studies Comparing Physiological, Perceptual and Performance Measures Between Treadmill and Overground Running,” Sports Medicine 49, no. 5 (2019): 763–782, 10.1007/s40279-019-01087-9. 30847825

[31] A. Mündermann , B. M. Nigg , D. J. Stefanyshyn , and R. N. Humble , “Development of a Reliable Method to Assess Footwear Comfort During Running,” Gait & Posture 16, no. 1 (2002): 38–45. 12127185 10.1016/s0966-6362(01)00197-7

[32] R. A. Robergs , D. Dwyer , and T. Astorino , “Recommendations for Improved Data Processing From Expired Gas Analysis Indirect Calorimetry,” Sports Medicine 40, no. 2 (2010): 95–111, 10.2165/11319670-000000000-00000. 20092364

[33] A. E. Jeukendrup and G. A. Wallis , “Measurement of Substrate Oxidation During Exercise by Means of Gas Exchange Measurements,” International Journal of Sports Medicine 26 (2005): S28–S37, 10.1055/s-2004-830512. 15702454

[34] M. R. Paquette , J. A. Melaro , R. Smith , and I. S. Moore , “Time to Stability of Treadmill Running Kinematics in Novel Footwear With Different Midsole Thickness,” Journal of Biomechanics 164 (2024): 111984. 38330884 10.1016/j.jbiomech.2024.111984

[35] T. J. van der Zee , P. Tecchio , D. Hahn , and B. J. Raiteri , “UltraTimTrack: A Kalman‐Filter‐Based Algorithm to Track Muscle Fascicles in Ultrasound Image Sequences,” PeerJ Computer Science 11 (2025): e2636. 10.7717/peerj-cs.2636 PMC11784871 39896012

[36] W. Hopkins , “A New View of Statistics,” accessed 6 February, 2025, https://www.sportsci.org/resource/stats/effectmag.html.

[37] G. T. Burns and D. P. Joubert , “Running Shoes of the Postmodern Footwear Era: A Narrative Overview of Advanced Footwear Technology,” International Journal of Sports Physiology and Performance 19, no. 10 (2024): 975–986. 39117307 10.1123/ijspp.2023-0446

[38] V. Rodrigo‐Carranza , W. Hoogkamer , J. M. González‐Ravé , and F. González‐Mohíno , “Relationship Between Advanced Footwear Technology Longitudinal Bending Stiffness and Energy Cost of Running,” Scandinavian Journal of Medicine & Science in Sports 34, no. 6 (2024): e14687. 38923087 10.1111/sms.14687

[39] N. Flores , N. Delattre , E. Berton , and G. Rao , “Does an Increase in Energy Return and/or Longitudinal Bending Stiffness Shoe Features Reduce the Energetic Cost of Running?,” European Journal of Applied Physiology 119 (2019): 429–439. 30470873 10.1007/s00421-018-4038-1

[40] M. Bertschy , H. Lino , L. Healey , et al., “Is Increasing the Effective Leg Length of a Human Runner Metabolically Beneficial?” bioRxiv: 2025.01.03.631222, 2025. 10.1242/jeb.250107 PMC12582409 40955759

[41] M. R. Shorten , “Energy Return in Footwear–Revisited,” Footwear Science 16, no. 3 (2024): 149–162.

[42] E. S. Matijevich , E. C. Honert , F. Yang , W. K. Lam , and B. M. Nigg , “Greater Foot and Footwear Mechanical Work Associated With Less Ankle Joint Work During Running,” Sports Biomechanics (2024): 1–19. 10.1080/14763141.2023.2296916 38164950

[43] J. R. Franz , C. M. Wierzbinski , and R. Kram , “Metabolic Cost of Running Barefoot Versus Shod: Is Lighter Better?,” Medicine and Science in Sports and Exercise 44, no. 8 (2012): 1519–1525. 22367745 10.1249/MSS.0b013e3182514a88

[44] D. P. Joubert and G. P. Jones , “A Comparison of Running Economy Across Seven Highly Cushioned Racing Shoes With Carbon‐Fibre Plates,” Footwear Science 14 (2022): 1–13.37701063

[45] Z. B. Barrons , J. W. Wannop , and D. J. Stefanyshyn , “The Influence of Footwear Midsole Thickness on Running Economy and Frontal Plane Ankle Stability,” Footwear Science 15, no. 3 (2023): 155–160.

[46] V. Rodrigo‐Carranza , F. González‐Mohíno , J. Santos‐Concejero , et al., “Influence of Shoe Mass on Performance and Running Economy in Trained Runners,” Frontiers in Physiology 11 (2020): 573660, 10.3389/fphys.2020.573660. 33071828 PMC7538857

[47] S. Cigoja , J. R. Fletcher , M. Esposito , D. J. Stefanyshyn , and B. M. Nigg , “Increasing the Midsole Bending Stiffness of Shoes Alters Gastrocnemius Medialis Muscle Function During Running,” Scientific Reports 11, no. 1 (2021): 749. 33436965 10.1038/s41598-020-80791-3 PMC7804138

[48] A. Fife , C. Ramsey , J. F. Esculier , and K. Hébert‐Losier , “How Do Runners Select Their Shoes? An In‐Store Experience,” Footwear Science 16 (2024): 1–199.

[49] O. N. Beck , P. R. Golyski , and G. S. Sawicki , “Adding Carbon Fiber to Shoe Soles May Not Improve Running Economy: A Muscle‐Level Explanation,” Scientific Reports 10, no. 1 (2020): 1–13. 33051532 10.1038/s41598-020-74097-7 PMC7555508

[50] M. Riedl , C. von Diecken , and O. Ueberschär , “One Shoe to Fit Them All? Effect of Various Carbon Plate Running Shoes on Running Economy in Male and Female Amateur Triathletes and Runners at Individual Training and Race Paces,” Applied Sciences 14, no. 24 (2024): 11535.

[51] O. N. Beck , J. Gosyne , J. R. Franz , and G. S. Sawicki , “Cyclically Producing the Same Average Muscle‐Tendon Force With a Smaller Duty Increases Metabolic Rate,” Proceedings. Biological Sciences 287, no. 1933 (2020): 20200431, 10.1098/rspb.2020.0431. 32811308 PMC7482283

[52] S. Kipp , R. Kram , and W. Hoogkamer , “Extrapolating Metabolic Savings in Running: Implications for Performance Predictions,” Frontiers in Physiology 10, no. 79 (2019): 79, 10.3389/fphys.2019.00079. 30804807 PMC6378703

