# Supplementary material for: The Mediating Effect of Running Biomechanics, Anthropometrics, Muscle Architecture, and Comfort on Running Economy Across Different Shoes
Source: Scand J Med Sci Sports. 2025 Jun 5;35(6):e70087. doi: 10.1111/sms.70087 (PMC12141809; doi:10.1111/sms.70087)
Supplement: Supplementary file 1 — Table S1. [file SMS-35-e70087-s001.docx]

**Supplemental file: Additional data**

**Brief description mechanical tests**

A free mass of 8.1 kg with a rounded pestle was dropped 30 times from a height of 50 mm on the heel. The average value of the last five impacts were used to determine the mechanical properties for each shoe from the force-displacement curve. The longitudinal bending stiffness was determined with a 3-point bending test. To this purpose, the shoe was placed on 2 support frames and an mechanical machine, aligned with the metatarsophalangeal joint, displaced the shoe while recording the force required to do so.

**Table S1 Pairwise comparisons of contact time between the five shoe models**

| **Shoe** | **Shoe** | **Marginal Mean ± SE** | **P-value** |
| --- | --- | --- | --- |
| ASICS Metaspeed Sky + | Kiprun KD900X LD | -4.59 ± 1.17 | <0.01* |
|  | Kiprun KS900 Light | -1.51 ± 1.17 | 0.20 |
|  | Own running shoe | -0.98 ± 1.21 | 0.42 |
|  | Saucony Fastwitch 9 | -1.40 ± 1.18 | 0.24 |
| Kiprun KD900X LD | Kiprun KS900 Light | 3.08 ± 1.17 | 0.01* |
|  | Own running shoe | 3.62 ± 1.21 | <0.01* |
|  | Saucony Fastwitch 9 | 3.19 ± 1.18 | <0.01* |
| Kiprun KS900 Light | Own running shoe | 0.54 ± 1.21 | 0.66 |
|  | Saucony Fastwitch 9 | 0.11 ± 1.18 | 0.92 |
| Own running shoe | Saucony Fastwitch 9 | -0.42 ± 1.22 | 0.73 |

Statistically significant differences) after decreasing the threshold value for statistical significance to *p*<0.0125 are indicated by asterisks (*).

**Table S2 Pairwise comparisons of flight time between the five shoe models**

| **Shoe** | **Shoe** | **Marginal Mean ± SE** | **P-value** |
| --- | --- | --- | --- |
| ASICS Metaspeed Sky + | Kiprun KD900X LD | 3.52 ± 1.27 | <0.01* |
|  | Kiprun KS900 Light | 1.96 ± 1.27 | 0.13 |
|  | Own running shoe | 1.37 ± 1.32 | 0.30 |
|  | Saucony Fastwitch 9 | 3.42 ± 1.28 | <0.01* |
| Kiprun KD900X LD | Kiprun KS900 Light | -1.57 ± 1.27 | 0.22 |
|  | Own running shoe | -2.15 ± 1.32 | 0.10 |
|  | Saucony Fastwitch 9 | -0.10 ± 1.29 | 0.94 |
| Kiprun KS900 Light | Own running shoe | -0.59 ± 1.32 | 0.66 |
|  | Saucony Fastwitch 9 | 1.46 ± 1.29 | 0.26 |
| Own running shoe | Saucony Fastwitch 9 | 2.05 ± 1.33 | 0.12 |

Statistically significant differences) after decreasing the threshold value for statistical significance to *p*<0.0125 are indicated by asterisks (*).

**Table S3 Pairwise comparisons of cadence between the five shoe models**

| **Shoe** | **Shoe** | **Marginal Mean ± SE** | **P-value** |
| --- | --- | --- | --- |
| ASICS Metaspeed Sky + | Kiprun KD900X LD | 0.69 ± 0.31 | 0.03 |
|  | Kiprun KS900 Light | 0.02 ± 0.31 | 0.95 |
|  | Own running shoe | -0.20 ± 0.32 | 0.37 |
|  | Saucony Fastwitch 9 | -0.79 ± 0.31 | 0.01 |
| Kiprun KD900X LD | Kiprun KS900 Light | -0.67 ± 0.31 | 0.03 |
|  | Own running shoe | -0.98 ± 0.32 | <0.01* |
|  | Saucony Fastwitch 9 | -1.48 ± 0.31 | <0.01* |
| Kiprun KS900 Light | Own running shoe | -0.31 ± 0.32 | 0.34 |
|  | Saucony Fastwitch 9 | -0.81 ± 0.31 | <0.01* |
| Own running shoe | Saucony Fastwitch 9 | -0.50 ± 0.31 | 0.12 |

Statistically significant differences) after decreasing the threshold value for statistical significance to *p*<0.0125 are indicated by asterisks (*).

**Table S4 Pairwise comparisons of duty factor between the five shoe models**

| **Shoe** | **Shoe** | **Marginal Mean ± SE** | **P-value** |
| --- | --- | --- | --- |
| ASICS Metaspeed Sky + | Kiprun KD900X LD | 0.70 ± 0.36 | 0.05 |
|  | Kiprun KS900 Light | 0.26 ± 0.36 | 0.46 |
|  | Own running shoe | 0.46 ± 0.36 | 0.20 |
|  | Saucony Fastwitch 9 | 0.40 ± 0.36 | 0.29 |
| Kiprun KD900X LD | Kiprun KS900 Light | -0.44 ± 0.35 | 0.21 |
|  | Own running shoe | -0.24 ± 0.35 | 0.50 |
|  | Saucony Fastwitch 9 | -0.31 ± 0.36 | 0.40 |
| Kiprun KS900 Light | Own running shoe | 0.20 ± 0.35 | 0.57 |
|  | Saucony Fastwitch 9 | 0.13 ± 0.36 | 0.72 |
| Own running shoe | Saucony Fastwitch 9 | -0.07 ± 0.36 | 0.86 |

Statistically significant differences) after decreasing the threshold value for statistical significance to *p*<0.0125 are indicated by asterisks (*).

**Table S5 Pairwise comparisons of overall comfort between the five shoe models**

| **Shoe** | **Shoe** | **Marginal Mean ± SE** | **P-value** |
| --- | --- | --- | --- |
| ASICS Metaspeed Sky + | Kiprun KD900X LD | -0.15 ± 0.32 | 0.64 |
|  | Kiprun KS900 Light | 0.14 ± 0.31 | 0.66 |
|  | Own running shoe | -0.91 ± 0.31 | <0.01* |
|  | Saucony Fastwitch 9 | 0.90 ± 0.32 | 0.01* |
| Kiprun KD900X LD | Kiprun KS900 Light | 0.28 ± 0.32 | 0.37 |
|  | Own running shoe | -0.77 ± 0.32 | 0.02 |
|  | Saucony Fastwitch 9 | 1.04 ± 0.32 | <0.01* |
| Kiprun KS900 Light | Own running shoe | -1.05 ± 0.31 | <0.01* |
|  | Saucony Fastwitch 9 | 0.76 ± 0.32 | 0.02 |
| Own running shoe | Saucony Fastwitch 9 | 1.81 ± 0.32 | <0.01* |

Statistically significant differences) after decreasing the threshold value for statistical significance to *p*<0.0125 are indicated by asterisks (*).

**Table S6 Pairwise comparisons of heel cushioning between the five shoe models**

| **Shoe** | **Shoe** | **Marginal Mean ± SE** | **P-value** |
| --- | --- | --- | --- |
| ASICS Metaspeed Sky + | Kiprun KD900X LD | -0.33 ± 0.37 | 0.37 |
|  | Kiprun KS900 Light | -0.09 ± 0.37 | 0.80 |
|  | Own running shoe | -0.67 ± 0.37 | 0.07 |
|  | Saucony Fastwitch 9 | 1.26 ± 0.37 | <0.01* |
| Kiprun KD900X LD | Kiprun KS900 Light | 0.24 ± 0.37 | 0.52 |
|  | Own running shoe | -0.34 ± 0.37 | 0.36 |
|  | Saucony Fastwitch 9 | 1.59 ± 0.37 | <0.01* |
| Kiprun KS900 Light | Own running shoe | -0.58 ± 0.37 | 0.12 |
|  | Saucony Fastwitch 9 | 1.35 ± 0.37 | <0.01* |
| Own running shoe | Saucony Fastwitch 9 | 1.93 ± 0.37 | <0.01* |

Statistically significant differences) after decreasing the threshold value for statistical significance to *p*<0.0125 are indicated by asterisks (*).

**Table S7 Pairwise comparisons of forefoot cushioning between the five shoe models**

| **Shoe** | **Shoe** | **Marginal Mean ± SE** | **P-value** |
| --- | --- | --- | --- |
| ASICS Metaspeed Sky + | Kiprun KD900X LD | -0.22 ± 0.40 | 0.58 |
|  | Kiprun KS900 Light | 0.77 ± 0.40 | 0.05 |
|  | Own running shoe | -0.17 ± 0.40 | 0.67 |
|  | Saucony Fastwitch 9 | 1.44 ± 0.40 | <0.01* |
| Kiprun KD900X LD | Kiprun KS900 Light | 1.00 ± 0.40 | 0.01 |
|  | Own running shoe | 0.05 ± 0.40 | 0.89 |
|  | Saucony Fastwitch 9 | 1.66 ± 0.40 | <0.01* |
| Kiprun KS900 Light | Own running shoe | -0.95 ± 0.40 | 0.02 |
|  | Saucony Fastwitch 9 | 0.66 ± 0.40 | 0.10 |
| Own running shoe | Saucony Fastwitch 9 | 1.61 ± 0.40 | <0.01* |

Statistically significant differences) after decreasing the threshold value for statistical significance to *p*<0.0125 are indicated by asterisks (*).

**Table S8 Pairwise comparisons of mediolateral control between the five shoe models**

| **Shoe** | **Shoe** | **Marginal Mean ± SE** | **P-value** |
| --- | --- | --- | --- |
| ASICS Metaspeed Sky + | Kiprun KD900X LD | 0.16 ± 0.37 | 0.66 |
|  | Kiprun KS900 Light | 0.76 ± 0.37 | 0.04 |
|  | Own running shoe | -1.93 ± 0.37 | <0.01* |
|  | Saucony Fastwitch 9 | -0.44 ± 0.37 | 0.24 |
| Kiprun KD900X LD | Kiprun KS900 Light | -0.93 ± 0.37 | 0.01 |
|  | Own running shoe | -2.10 ± 0.37 | <0.01* |
|  | Saucony Fastwitch 9 | -0.60 ± 0.37 | 0.11 |
| Kiprun KS900 Light | Own running shoe | -1.17 ± 0.37 | <0.01* |
|  | Saucony Fastwitch 9 | 0.32 ± 0.37 | 0.38 |
| Own running shoe | Saucony Fastwitch 9 | 1.50 ± 0.37 | <0.01* |

Statistically significant differences) after decreasing the threshold value for statistical significance to *p*<0.0125 are indicated by asterisks (*).

**Table S9 Pairwise comparisons of arc height comfort between the five shoe models**

| **Shoe** | **Shoe** | **Marginal Mean ± SE** | **P-value** |
| --- | --- | --- | --- |
| ASICS Metaspeed Sky + | Kiprun KD900X LD | -0.20 ± 0.35 | 0.57 |
|  | Kiprun KS900 Light | -0.26 ± 0.35 | 0.46 |
|  | Own running shoe | -1.03 ± 0.35 | <0.01* |
|  | Saucony Fastwitch 9 | 0.54 ± 0.35 | 0.13 |
| Kiprun KD900X LD | Kiprun KS900 Light | -0.06 ± 0.35 | 0.86 |
|  | Own running shoe | -0.82 ± 0.35 | 0.02 |
|  | Saucony Fastwitch 9 | 0.74 ± 0.36 | 0.04 |
| Kiprun KS900 Light | Own running shoe | -0.76 ± 0.35 | 0.03 |
|  | Saucony Fastwitch 9 | 0.80 ± 0.35 | 0.03 |
| Own running shoe | Saucony Fastwitch 9 | -1.57 ± 0.35 | <0.01* |

Statistically significant differences) after decreasing the threshold value for statistical significance to *p*<0.0125 are indicated by asterisks (*).

**Table S10 Pairwise comparisons of heel cup fit between the five shoe models**

| **Shoe** | **Shoe** | **Marginal Mean ± SE** | **P-value** |
| --- | --- | --- | --- |
| ASICS Metaspeed Sky + | Kiprun KD900X LD | -0.22 ± 0.35 | 0.53 |
|  | Kiprun KS900 Light | -0.76 ± 0.34 | 0.03 |
|  | Own running shoe | -1.49 ± 0.34 | <0.01* |
|  | Saucony Fastwitch 9 | -0.10 ± 0.35 | 0.76 |
| Kiprun KD900X LD | Kiprun KS900 Light | -0.55 ± 0.35 | 0.12 |
|  | Own running shoe | -1.27 ± 0.35 | <0.01* |
|  | Saucony Fastwitch 9 | 0.11 ± 0.35 | 0.75 |
| Kiprun KS900 Light | Own running shoe | -0.72 ± 0.34 | 0.04 |
|  | Saucony Fastwitch 9 | 0.66 ± 0.35 | 0.06 |
| Own running shoe | Saucony Fastwitch 9 | -1.38 ± 0.35 | <0.01* |

Statistically significant differences) after decreasing the threshold value for statistical significance to *p*<0.0125 are indicated by asterisks (*).

**Table S11. Correlation coefficients between shoe characteristics and running economy and overall comfort, heel cushioning, and medio-lateral stability**

| **Shoe characteristic** | **Correlation coefficient RE** | **Correlation coefficient overall comfort** | **Correlation coefficient heel cushioning** | **Correlation coefficient medio-lateral stability** |
| --- | --- | --- | --- | --- |
| Mass (g) | 0.46 | 0.55 | -0.56 | 0.40 |
| Drop (mm) | 0.59 | -0.05 | -0.41 | 0.88 |
| Longitudinal bending stiffness (N.mm^-1^) | -0.92 | 0.57 | -0.30 | -0.95 |
| Stack height (mm) | -0.59 | 0.96 | -0.83 | -0.53 |
| Peak force (N) | 0.62 | -0.96 | 0.67 | 0.70 |
| Max displacement (mm) | -0.55 | 0.91 | -0.43 | -0.81 |
| Energy Input (J) | 0.07 | 0.87 | -0.42 | -0.29 |
| Energy Return (J) | -0.94 | 0.68 | -0.58 | -0.80 |
| Energy Loss (J) | 0.99 | -0.44 | 0.48 | 0.74 |
| Energy return (%) | -0.98 | 0.55 | -0.54 | -0.76 |
| Heel compression stiffness (N∙mm^-1^) | 0.58 | -0.96 | 0.63 | 0.71 |
